# Improved Coarray Interpolation Algorithms with Additional Orthogonal Constraint for Cyclostationary Signals

**DOI:** 10.3390/s18010219

**Published:** 2018-01-14

**Authors:** Jinyang Song, Feng Shen

**Affiliations:** 1 College of Automation, Harbin Engineering University, No. 145 Nantong Street, Harbin 150001, China; songjy@hrbeu.edu.cn; 2 School of Electrical Engineering & Automation, Harbin Institute of Technology, No. 92 Xidazhi Street, Harbin 150006, China

**Keywords:** coarray interpolation, cyclostationarity, (conjugate) correlation subspaces, coprime array, orthogonal constraint, Toeplitz completion, Hankel completion

## Abstract

Many modulated signals exhibit a cyclostationarity property, which can be exploited in direction-of-arrival (DOA) estimation to effectively eliminate interference and noise. In this paper, our aim is to integrate the cyclostationarity with the spatial domain and enable the algorithm to estimate more sources than sensors. However, DOA estimation with a sparse array is performed in the coarray domain and the holes within the coarray limit the usage of the complete coarray information. In order to use the complete coarray information to increase the degrees-of-freedom (DOFs), sparsity-aware-based methods and the difference coarray interpolation methods have been proposed. In this paper, the coarray interpolation technique is further explored with cyclostationary signals. Besides the difference coarray model and its corresponding Toeplitz completion formulation, we build up a sum coarray model and formulate a Hankel completion problem. In order to further improve the performance of the structured matrix completion, we define the spatial spectrum sampling operations and the derivative (conjugate) correlation subspaces, which can be exploited to construct orthogonal constraints for the autocorrelation vectors in the coarray interpolation problem. Prior knowledge of the source interval can also be incorporated into the problem. Simulation results demonstrate that the additional constraints contribute to a remarkable performance improvement.

## 1. Introduction

Direction-of-arrival (DOA) estimation has been a popular research field in array processing for several decades. This problem aims at retrieving the directional information of sources from the array of received signals, and plays an important role in a variety of practical scenarios [1,2,3]. Conventional DOA estimation methods such as multiple signal classification (MUSIC) [4] and estimation of signal parameters via rotational invariance technique (ESPRIT) [5] basically rely on the spatial properties of the signals impinging on the antenna array, while ignoring the temporal and frequency properties. However, many modulated signals used in communication, radar, and sonar systems exhibit a cyclostationarity property. These cyclostationary signals are not periodic with respect to time, but their statistical characteristics vary periodically with time. This property can be exploited to effectively eliminate interference and background noise [6,7,8,9]. The cyclostationarity-based DOA estimation methods were started by Gardner in [6,7], and the proposed cyclic MUSIC and cyclic ESPRIT algorithms used a cyclic correlation matrix (CCM) in place of the zero-lag covariance matrix. An extended cyclic MUSIC was proposed in [10]; besides the CCM, an additional conjugate cyclic correlation matrix (CCCM) was also utilized to obtain an increase in degree-of-freedom from O(N) to O(2N−1). All the mentioned methods are proposed for uniform linear arrays (ULA), and fail to estimate the DOAs when the number of sources is larger than the DOFs of the ULA. Therefore, the main task in this paper is to integrate the cyclostationarity property with the spatial domain—which means that the second-order statistics incorporated with the cyclic frequency and the time lags are utilized, instead of the conventional covariance that only contains spatial information—and to improve the performance of DOA estimation under the circumstance that the sources are much more numerous than the sensors.

In order to estimate the DOAs of as many signals as possible under a given number of sensors, many sparse linear array structures have been proposed. The traditional one is the minimum redundancy array (MRA), which can generate consecutive virtual apertures by utilizing the coarray information [11,12]. However, the sensor locations are determined by exhaustive searching and no general expressions are available for the locations of the physical sensors in an MRA. This leads to an intensive investigation of the sparse linear arrays, such as the (super) nested arrays [13,14] and the (generalized) coprime arrays [15,16], which have a more concise and flexible geometry for sparse array configuration. These sparse arrays, consisting of two uniform linear arrays with different interelement spacing, open a new approach to array processing. They can resolve $O(N^{2})$ sources from merely N sensors. One distinctive advantage of the coprime array is that a negligible mutual coupling effect is introduced because the interelement spacing of the coprime array is several times longer than the half-wavelength, which makes it more attractive than the nested array. Intrinsically, these sparse arrays all intend to generate consecutive virtual apertures by exploiting the difference coarray. The arrays with a filled coarray are called fully augmentable arrays and when there are holes in their coarray, as in the case of coprime arrays, they are called partially augmentable arrays [17]. Three kinds of algorithms have been developed for these two types of augmentable arrays.

The first kind used compressive sensing (CS)-inspired $l_{1}$ norm minimization techniques [18,19,20] by assuming a sparse representation of the incoming DOAs on a prespecified discrete spatial grid. However, the CS-based methods suffer from basis mismatch effects since the true DOAs are unlikely to lie on the prespecified grid, no matter how fine it is chosen to be [21]. The second techniques are the spatial smoothing (SS) algorithms, which can construct a positive definite matrix in the difference coarray domain that contains noise subspace information. Once the spatially smoothed matrix is obtained as in [15,22] or by the direct coarray augmented method [23], the MUSIC method [22] and the ESPRIT method [24] algorithms can be used to perform the DOA estimation. An additional version of the smoothed matrix is proposed in [25], which is optimized through low-rank denoising to avoid the estimation of source numbers. The main limitation of these SS algorithms is that they can only utilize the consecutive virtual lags of the coarray; when there are holes as in the partially augmentable arrays, the lags out of the consecutive part of the coarray are ignored. In order to utilize the full coarray information, the Hermitian Toeplitz matrix completion-based array interpolation techniques were proposed [17,26]. Benefitting from the development of matrix completion theory [27,28], the simple nuclear norm minimization problem can be formulated to recover a low-rank matrix with small set of entries. Thus, the holes in the coarray can be filled via matrix completion. A few papers have taken into consideration the perturbation effect caused by the finite number of snapshots—an uncertainty is modelled into the matrix completion problem when assigning the existing values to their corresponding locations [29,30]. However, the error bound is set blindly and an improper setting may deteriorate the performance of the matrix completion. Moreover, [29] solved two sequential convex optimization problems, which was time-consuming and can be replaced by a modern structured matrix completion algorithm [31], while [30] utilized the redundant sample covariance to optimize the target low-rank matrix and did not make full use of the difference coarray information, leaving more entries in the matrix to be filled.

In this paper, we explore the coarray interpolation techniques with cyclostationary signals. Firstly, we use the cyclic correlation matrix and the conjugate cyclic correlation matrix as the second-order statistics and build up the difference coarray and the sum coarray models. Then, the spatial spectrum sampling operations and the derivative (conjugate) correlation subspaces are defined, and we demonstrate that the vectorization of the CCM and the CCCM lie in the correlation subspace and the conjugate correlation subspace, respectively. With the relationship between the vectorization of the sampling correlation matrices (the CCM and the CCCM) and their corresponding autocorrelation vectors in the coarray domains, we construct orthogonal constraints for the autocorrelation vectors. Moreover, prior knowledge of the source interval can be incorporated into the orthogonal constraints. Finally, we formulate a Toeplitz completion problem for the difference coarray model and a Hankel completion problem for the sum coarray model, and integrate the orthogonal constraints into the structured matrix completion problems. Numerical results demonstrate the superior performance of the proposed algorithms.

The main contribution of this paper can be summarized as follows:

- We investigate the array interpolation techniques with the cyclostationary signals, building up the difference coarray model and the sum coarray model by utilizing the structure of the CCM and CCCM in a coprime array, and then formulate the array interpolation as a Toeplitz completion problem and a Hankel completion problem.
- We define the spatial spectrum sampling operations, and prove that they can extract the entire power spectrum associated with the CCM and the CCCM. After vectorization of the spatial sampling operations, we show that the vectorization of the sampling correlation matrices lies in the subspaces, which are called the (conjugate) correlation subspaces.
- We build orthogonal constraints for the autocorrelation vectors in the coarray domains according to the (conjugate) correlation subspaces. Prior knowledge of the source interval can be incorporated into the orthogonal constraints, which can help to improve the performance of the structured matrix completion remarkably.

The rest of this paper is organized as follows. In Section 2, we introduce the cyclostationary signal model and build up the difference coarray and sum coarray models based on the coprime array. In Section 3, the spatial spectrum operations and the derivative (conjugate) correlation subspaces are defined; then, the orthogonal constraints—which can incorporate the prior knowledge of the source interval—are constructed and integrated into the structured matrix completion problems. Section 4 presents the simulation results for comparison, and conclusions are drawn in Section 5.

Notation: Throughout this paper, scalars, vectors, matrices, and sets are denoted by lowercase letters, lowercase letters in boldface, uppercase letters in boldface, and letters in blackboard, respectively. The superscripts ∗, T, and H denote the complex conjugate, the transpose, and the complex conjugate transpose. The Moore–Penrose pseudoinverse is denoted with superscript †. vec(·) and E(·) represent the vectorization and expectation operations. The symbol ⊗ denotes the Kronecker product. |A| denotes the cardinality of a set A. ${\left[A\right]}_{i,j}$ indicates the (i,j)th entry of A. The triangle bracket ${\langle x_{S}\rangle }_{n}$ represents the value corresponding to the support n∈S.

## 2. Cyclostationary Signal Model and the Coprime Coarray Model

In this section, we firstly introduce the cyclostationary signal model received by the coprime linear array, and then construct the coarray models based on the structure of the CCM and the CCCM.

### 2.1. Cyclostationary Signal Model

For vector s(t) formed by D cyclostationary signals, the CCM $R_{ss}^{\alpha }(\tau )$ and the CCCM $R_{ss^{*}}^{\alpha }(\tau )$ are defined as

(1) $$R_{ss}^{\alpha }(\tau )={\langle E[s(t+\tau /2)s^{H}(t-\tau /2)]e^{-j2\pi \alpha t}\rangle }_{t}$$

(2) $$R_{ss^{*}}^{\alpha }(\tau )={\langle E[s(t+\tau /2)s^{T}(t-\tau /2)]e^{-j2\pi \alpha t}\rangle }_{t}$$

where ${\langle \cdot \rangle }_{t}$ denotes infinite-time averaging with respect to t. Then, $s{\left(t\right)}_{k}$ (k=1,2,…D) is said to be cyclostationary if ${\left[R_{ss}^{\alpha }(\tau )\right]}_{k,k}$ or ${\left[R_{ss^{*}}^{\alpha }(\tau )\right]}_{k,k}$ does not equal zero at frequency α for some lag parameter τ. The value of the cyclic frequency α is related to the carrier frequency and the baud rate of the signal—it is usually integer multiples of the carrier frequency or the baud rate. Some cyclostationary signals may contain both nonzero cyclic correlation and nonzero conjugate cyclic correlation.

Consider D narrowband sources, exhibiting second-order cyclostationarity with a common cycle α, impinging on the antenna array, which contains P physical sensors with the pth sensor located at $z_{p}d$, where $z_{p}$ is an integer and d=λ/2 (λ being the carrier wavelength of the sources). The signal received on the pth sensor is

(3) $$x_{p}(t)=\sum_{k=1}^{D}s_{k}(t)e^{j2\pi (f_{c}/c)z_{p}d\sin\theta_{k}}+n_{p}(t)$$

where $s_{k}(t)$ is the kth zero-mean desired signal from direction $\theta_{k}$ and $n_{p}(t)$ is the zero-mean additive Gaussian white noise at the pth sensor. There are two assumptions that must hold: (a) the impinging sources are mutually cyclically uncorrelated, which means $R_{ss}^{\alpha }(\tau )$ and $R_{ss^{*}}^{\alpha }(\tau )$ are diagonal matrices; and (b) the sources are uncorrelated with the noise. Under assumptions (a) and (b), the cyclic correlation function and the conjugate cyclic correlation function between the pth and the qth sensor are given by

(4) $$R_{x_{p}x_{q}}^{\alpha }(\tau )={\langle E[x_{p}(t+\tau /2)x_{q}^{*}(t-\tau /2)]e^{-j2\pi \alpha t}\rangle }_{t}=\sum_{k=1}^{D}R_{s_{k}s_{k}}^{\alpha }(\tau )a_{p}(\theta_{k})a_{q}^{*}(\theta_{k})$$

(5) $$R_{x_{p}x_{q}^{*}}^{\alpha }(\tau )={\langle E[x_{p}(t+\tau /2)x_{q}(t-\tau /2)]e^{-j2\pi \alpha t}\rangle }_{t}=\sum_{k=1}^{D}R_{s_{k}s_{k}^{*}}^{\alpha }(\tau )a_{p}(\theta_{k})a_{q}(\theta_{k})$$

where $\alpha (\theta_{k})=[a_{1}(\theta_{k}),a_{2}(\theta_{k}),\ldots a_{P}(\theta_{k})]$ is the steering vector, whose pth element is defined as $a_{p}(\theta_{k})=e^{j2\pi (f_{c}/c)z_{p}d\sin(\theta_{k})}$. Therefore, the P×P CCM and the CCCM can be constructed with the elements for all indices p and q in Equations (4) and (5) as follows:

(6) $$R_{xx}^{\alpha }(\tau )=\sum_{k=1}^{D}R_{s_{k}s_{k}}^{\alpha }(\tau )\alpha (\theta_{k})\alpha^{H}(\theta_{k}),$$

(7) $$R_{{\mathrm{xx}}^{*}}^{\alpha }(\tau )=\sum_{k=1}^{D}R_{s_{k}s_{k}^{*}}^{\alpha }(\tau )\alpha (\theta_{k})\alpha^{T}(\theta_{k}).$$

In practice, only a finite number of snapshots are available. Let $\tilde{x}(t)$ denote the snapshots; the sampling correlation matrices, namely, the CCM and the CCCM, are obtained by

(8) $${\tilde{R}}_{xx}^{\alpha }(\tau )=\frac{1}{N}\sum_{n=1}^{N}\tilde{x}(t_{n}+\tau /2){\tilde{x}}^{H}(t_{n}-\tau /2)e^{-j2\pi \alpha t},$$

(9) $${\tilde{R}}_{{\mathrm{xx}}^{*}}^{\alpha }(\tau )=\frac{1}{N}\sum_{n=1}^{N}\tilde{x}(t_{n}+\tau /2){\tilde{x}}^{T}(t_{n}-\tau /2)e^{-j2\pi \alpha t}.$$

### 2.2. Coprime Coarray Model

In order to generate the augmented array, we firstly define the difference coarray and the sum coarray as the following:

**Definition** **1.** *Let* $S=\{z_{p},1\leq p\leq P\}$ *denote the set of sensor positions of a sparse array (normalized with respect to* d*); then, the difference coarray* ${\mathbb{C}}_{d}$ *and the sum coarray* ${\mathbb{C}}_{s}$ *are defined respectively as*

(10) $${\mathbb{C}}_{d}=\{z_{p}-z_{q}|z_{p},z_{q}\in S\},$$

(11) $${\mathbb{C}}_{s}=\{z_{p}+z_{q}|z_{p},z_{q}\in S\}.$$

Then, a steering vector associated with the coarray can be formed using ${\left[\alpha_{\mathbb{C}d(s)}(\theta_{k})\right]}_{m}=e^{j2\pi (f_{c}/c)z_{m}d\sin(\theta_{k})}$, $z_{m}\in {\mathbb{C}}_{d(s)}$. The autocorrelation vectors at lag set ${\mathbb{C}}_{d(s)}$ can be represented by $r_{\mathbb{C}d(s)}=\sum_{k=1}^{D}R_{s_{k}s_{k}^{\left(*\right)}}^{\alpha }(\tau )\alpha_{\mathbb{C}d(s)}(\theta_{k})$, and exhibit a linear relationship with the vectorization of the CCM and the CCCM given by

(12) $$J_{d}r_{\mathbb{C}d}=\mathrm{vec}(R_{xx}^{\alpha }(\tau )),$$

(13) $$J_{s}r_{\mathbb{C}s}=\mathrm{vec}(R_{xx^{*}}^{\alpha }(\tau )).$$

$J_{d}$ and $J_{s}$ are the transform matrices, which are defined as in [32]:

**Definition** **2.** *The binary matrices* $J_{d(s)}$ *are of dimension* $\left|{\mathbb{C}}_{d(s)}\right|\times \left|S^{2}\right|$*. The columns of* $J_{d(s)}$ *satisfy* ${\langle J_{d(s)}\rangle }_{:,m}=\mathrm{vec}(I(m))$ *for* $m\in {\mathbb{C}}_{d(s)}$ *, where* $I(m)\in {\left\{0,1\right\}}^{\left|S\right|\times \left|S\right|}$ *is an indicator function given by*

(14) $$I{\left(m\right)}_{n1,n2}=\left\{ \begin{array}{ccc} 1, & \mathrm{if}\;n1-n2=m & \mathrm{for}\;\mathrm{difference}\;\mathrm{coarray}\\ 1, & \mathrm{if}\;n1+n2=m & \mathrm{for}\;\mathrm{sum}\;\mathrm{coarray}\\ 0, & \mathrm{otherwise}. & \end{array}\right.$$

Let $r_{\mathbb{C}d(s)}$ be the signal received at a virtual sensor array with sensor positions given by ${\mathbb{C}}_{d(s)}$. If a consecutive virtual aperture can be formed by using the difference coarray or the sum coarray definition, we can resort to spatial smoothing techniques [15,23] to recover their ranks, due to the fact that $r_{\mathbb{C}d(s)}$ is a rank one vector. Otherwise, a partially augmentable array will be formed, and, in order to utilize the full coarray information, we intend to fill the holes within the augmented uniform linear arrays (ULA) $V_{d(s)}$, defined as follows:

**Definition** **3.** *Let* $V_{d(s)}$ *be the smallest ULA containing* ${\mathbb{C}}_{d(s)}$ *such that $V_{d(s)}=\{m|\min({\mathbb{C}}_{d(s)})\leq m\leq \max({\mathbb{C}}_{d(s)})\}$.*

**Figure 1:**
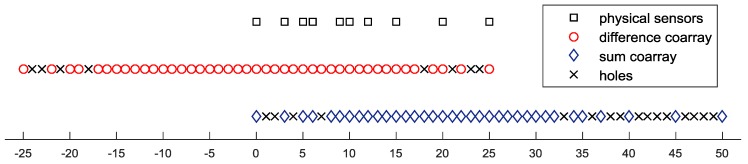
The configuration of a coprime array and its corresponding coarrays.

To give an intuitive impression of these configurations of the coprime array, we give an example by assuming a coprime array with physical sensor positions at S={0,3,5,6,9,10,12,15,20,25}. The difference coarray and sum coarray are shown in Figure 1 with red circles and blue diamonds, respectively. Holes within the coarrays are marked with crosses.

## 3. (Conjugate) Correlation Subspaces and the Proposed Coarray Interpolation Algorithms

The coarray interpolation techniques are proposed on the basis of matrix completion theory, and the Hermitian Toeplitz structure revealed in [23] is enforced on the matrix under estimation for regulation. In order to further improve the performance of the coarray interpolation, we explore the additional structure of the autocorrelation vectors, proposing definitions of spatial spectrum sampling operations and the derivative (conjugate) correlation subspaces, and then integrate the derivative orthogonal constraints into the coarray interpolation algorithms.

### 3.1. (Conjugate) Correlation Subspaces

Inspired by the compressive covariance sensing [33] that the array covariance matrix has a sparse representation in a dictionary constructed from the steering vectors, and the method for constructing orthogonal constraints by utilizing the spatial feature [34,35,36], we consider constructing a subspace in which the correlation matrix lies so that an orthogonal constraint can be constructed for the correlation matrix. Firstly, we give the definitions of the spatial spectrum sampling operations.

**Definition** **4.** *For a linear array of* P *physical sensors, and* D *cyclostationary signals impinging on the antenna array from directions* $\theta_{k}$ *, the spatial spectrum sampling operations for the CCM* $SS(R_{xx}^{\alpha }(\tau ))$ *and the CCCM* $CSS(R_{xx^{*}}^{\alpha }(\tau ))$ *are defined respectively as follows:*

(15) $$SS(R_{xx}^{\alpha }(\tau ))=\int_{\theta_{i}}\alpha (\theta_{i})\alpha^{H}(\theta_{i})\sum_{k=1}^{D}R_{s_{k}s_{k}}^{\alpha }(\tau )\alpha (\theta_{k})\alpha^{H}(\theta_{k})\alpha (\theta_{i})\alpha^{H}(\theta_{i})d\theta_{i}/P^{2},$$

(16) $$CSS(R_{xx^{*}}^{\alpha }(\tau ))=\int_{\theta_{i}}\alpha (\theta_{i})\alpha^{H}(\theta_{i})\sum_{k=1}^{D}R_{s_{k}s_{k}}^{\alpha }(\tau )\alpha (\theta_{k})\alpha^{T}(\theta_{k})\alpha^{*}(\theta_{i})\alpha^{T}(\theta_{i})d\theta_{i}/P^{2}.$$

We observe that for an ULA with P physical sensors, if P is large enough, the P-points averaged inner product of the two steering vectors $\alpha^{H}(\theta_{i})\alpha (\theta_{k})/P$ ($\theta_{i},\theta_{k}\in [-\pi /2,\pi /2]$) can be approximated by a Kronecker delta function, i.e.,

(17) $$\alpha^{H}(\theta_{i})\alpha (\theta_{k})/P\approx \delta_{i,k}=\left\{ \begin{array}{ll} 1, & i=k\\ 0, & i\neq k \end{array}\right..$$

**Proof.**  See Appendix A

When the nonuniform linear array is a coprime array, the inner product $\alpha^{H}(\theta_{i})\alpha (\theta_{k})/P$ with $\theta_{i}=\theta_{k}$ is much larger than the other values when $\theta_{i}\neq \theta_{k}$, and we can still approximate it with a Kronecker delta function. Thus, Equations (15) and (16) can be written as

(18) $$SS(R_{xx}^{\alpha }(\tau ))=\sum_{k=1}^{D}R_{s_{k}s_{k}}^{\alpha }(\tau )\int_{\theta_{i}}\alpha (\theta_{i})\delta_{i,k}\delta_{k,i}\alpha^{H}(\theta_{i})d\theta_{i}=R_{xx}^{\alpha }(\tau ),$$

(19) $$CSS(R_{xx^{*}}^{\alpha }(\tau ))=\sum_{k=1}^{D}R_{s_{k}s_{k}^{*}}^{\alpha }(\tau )\int_{\theta_{i}}\alpha (\theta_{i})\delta_{i,k}\delta_{k,i}^{*}\alpha^{T}(\theta_{i})d\theta_{i}=R_{xx^{*}}^{\alpha }(\tau ).$$

It can be seen from (18) and (19) that the integral parts will be nonzero only when i=k, and the final results of (18) and (19) will equal to $R_{xx}^{\alpha }(\tau )$ and $R_{xx^{*}}^{\alpha }(\tau )$, respectively, which means that the spatial spectrum sampling operations can extract the entire power spectrum associated with the CCM and the CCCM. With the observations of (18) and (19), we then vectorize (15) and (16), and utilize the following property [37]:

(20) $$\mathrm{vec}(ABC)=(C^{T}⊗A)\mathrm{vec}(B).$$

We thus obtain the following equations:

(21) $$\mathrm{vec}(R_{xx}^{\alpha }(\tau ))=1/P^{2}\int_{\theta_{i}}\left[\alpha^{*}(\theta_{i})\alpha^{T}(\theta_{i}\right)]⊗[\alpha (\theta_{i})\alpha^{H}(\theta_{i})]d\theta_{i}\mathrm{vec}(R_{xx}^{\alpha }(\tau )),$$

(22) $$\mathrm{vec}(R_{xx^{*}}^{\alpha }(\tau ))=1/P^{2}\int_{\theta_{i}}\left[\alpha (\theta_{i})\alpha^{H}(\theta_{i}\right)]⊗[\alpha (\theta_{i})\alpha^{H}(\theta_{i})]d\theta_{i}\mathrm{vec}(R_{xx^{*}}^{\alpha }(\tau )).$$

It can be seen from (21) and (22) that the vectors ($\mathrm{vec}(R_{xx}^{\alpha }(\tau ))$, $\mathrm{vec}(R_{xx^{*}}^{\alpha }(\tau ))$) are equal to themselves after being multiplied by the matrix (not an identical matrix) constructed by the integral with respect to $\theta_{i}$. We can draw a conclusion that the vectorization of the correlation matrices is in the column subspaces of these matrices. We refer to these matrices as the correlation subspace matrix $M_{CS}$ and the conjugate correlation subspace matrix $M_{CCS}$ with the following definitions:

**Definition** **5.** *The correlation subspace matrix* $M_{CS}$ *and the conjugate correlation subspace matrix* $M_{CCS}$ *are* $P^{2}\times P^{2}$ *matrices with*

(23) $$M_{CS}=\int_{\theta_{i}}\left[\alpha^{*}(\theta_{i})\alpha^{T}(\theta_{i}\right)]⊗[\alpha (\theta_{i})\alpha^{H}(\theta_{i})]d\theta_{i},$$

(24) $$M_{CCS}=\int_{\theta_{i}}\left[\alpha (\theta_{i})\alpha^{H}(\theta_{i}\right)]⊗[\alpha (\theta_{i})\alpha^{H}(\theta_{i})]d\theta_{i}.$$

**Figure 2:**
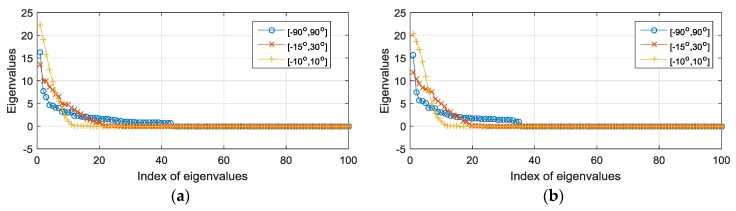
The dispersion of the eigenvalues for distinctive source intervals. (**a**) The eigenvalues of the correlation subspace matrix; (**b**) the eigenvalues of the conjugate correlation subspaces.

The integral interval can be [−π/2,π/2] or can be set according to prior knowledge about the sources. The vectorization of the CCM and the CCCM is in the column subspaces of $M_{CS}$ and $M_{CCS}$, respectively. Since $M_{CS}$ and $M_{CCS}$ are low-rank matrices, we can simplify the representations of the subspaces by using the eigenvectors associated with the positive eigenvalues, denoted by $Q_{CS}$ and $Q_{CCS}$. These subspaces are dependent on the array configuration and prior knowledge about the sources. The dimensions of these subspaces are determined by the positive numbers of the eigenvalues of the (conjugate) correlation matrices. The eigenvalues of the (conjugate) correlation matrices are shown in Figure 2. We take the coprime array with the configuration illustrated in Figure 1 as an example.

As shown in Figure 2, when we have no prior knowledge about the sources and the integral interval is set to [−π/2,π/2], the numbers of the positive eigenvalues of the correlation subspace matrix and the conjugate correlation subspace matrix are 43 and 35, respectively, which coincide with $\left|{\mathbb{C}}_{d}\right|$ and $\left|{\mathbb{C}}_{s}\right|$. When a shorter source interval is available, we can use less eigenvectors to construct the subspaces, but the exact dimension can only be determined by numerical tests.

After the subspaces are specified, we can construct constraints for the sampling correlation matrices according to the fact that the vectorization of the (conjugate) correlation matrices is orthogonal to the complementary subspaces of $Q_{CS}$ and $Q_{CCS}$, so we have

(25) $$(I-Q_{CS}Q_{CS}^{†})vec(R_{xx}^{\alpha }(\tau ))=0,$$

(26) $$(I-Q_{CCS}Q_{CCS}^{†})vec(R_{xx^{*}}^{\alpha }(\tau ))=0,$$

where $I-Q_{CS}Q_{CS}^{†}$ and $I-Q_{CCS}Q_{CCS}^{†}$ are the projection matrices on the orthogonal subspaces. According to (25) and (26), the denoised correlation matrices can be obtained by projecting the sampling correlation matrices to their individual subspaces such that the estimation error caused by the finite length of the snapshots can be eliminated. Here, we emphasis the construction of constraints for the structured matrix completion problems rather than the operations for sampling correlation matrix denoising. Thus, we transform these constraints into the coarray domains according to the relationship between the autocorrelation vectors and the vectorization of the (conjugate) correlation matrices shown in (12) and (13), obtaining

(27) $$(I-Q_{CS}Q_{CS}^{†})J_{d}r_{\mathbb{C}d}=0,$$

(28) $$(I-Q_{CCS}Q_{CCS}^{†})J_{s}r_{\mathbb{C}s}=0.$$

### 3.2. Coarray Interpolation Algorithms

For the coprime array, the increased DOFs are achieved by utilizing the coarray information. When there are holes in the coarray, the lags out of the consecutive part of the coarray cannot be involved in implementing the DOA estimation. In order to reduce the estimation error and increase the DOFs, coarray interpolation algorithms are proposed which can utilize all the coarray information. It is based on the results of the direct coarray augmentation [23] method, which can obtain the same noise subspace as the spatial smoothing method [15,22] by rearranging the autocorrelation vector to form a Hermitian Toeplitz matrix. As to the holes in the coarray ${\mathbb{C}}_{d(s)}$, they can be filled based on the property that the matrix to be recovered has low-rank terms, related to the signal components. It is the typical form of matrix completion, which can recover a low-rank matrix via solving a nuclear norm minimization problem.

In the context of coarray interpolation for cyclostationary signals, we can formulate a Toeplitz completion problem for the difference coarray model according to the fact that the CCM has a Toeplitz structure, as shown in (6). We do not require it to be a Hermitian matrix due to the finite snapshot effect with the cyclostationary signals. The autocorrelation vector on the uniform grid $V_{d}$ is denoted by $r_{Vd}$, which can be considered the antidiagonal elements of the Toeplitz matrix (denoted by $T(r_{Vd})$). The sampling autocorrelation vector on the nonuniform grid ${\mathbb{C}}_{d}$ is denoted by ${\tilde{r}}_{\mathbb{C}d}$, which is obtained from $J_{d}^{†}\mathrm{vec}({\tilde{R}}_{xx}^{\alpha }(\tau ))$ according to Equation (12). In order to integrate the orthogonal constraint (27) into the Toeplitz completion problem, we start with the autocorrelation vector denoising problem:

(29) $$\begin{array}{ll} \min & {\left\|{\langle r_{Vd}\rangle }_{\mathbb{C}d}-{\tilde{r}}_{\mathbb{C}d}\right\|}_{2}\\ s.t. & (I-Q_{CS}Q_{CS}^{†})J_{d}{\langle r_{Vd}\rangle }_{\mathbb{C}d}=0. \end{array}$$

Then, we relax the minimization term with ${\langle r_{Vd}\rangle }_{\mathbb{C}d}-{\tilde{r}}_{{\mathbb{C}}_{d}}=\varepsilon$, where ε is the error term. Equation (29) is equivalent to

(30) $$\begin{array}{ll} \min & {\left\|\varepsilon \right\|}_{2}\\ s.t. & {\langle r_{Vd}\rangle }_{\mathbb{C}d}-{\tilde{r}}_{\mathbb{C}d}=\varepsilon \\ & (I-Q_{CS}Q_{CS}^{†})J_{d}{\langle r_{Vd}\rangle }_{\mathbb{C}d}=0. \end{array}$$

Finally, we incorporate (30) into the nuclear norm minimization problem, which results in

(31) $$\begin{array}{ll} \min & {\left\|T(r_{Vd})\right\|}_{*}+\tau {\left\|\varepsilon \right\|}_{2}\\ s.t. & {\langle r_{Vd}\rangle }_{\mathbb{C}d}-{\tilde{r}}_{\mathbb{C}d}=\varepsilon \\ & (I-Q_{CS}Q_{CS}^{†})J_{d}{\langle r_{Vd}\rangle }_{\mathbb{C}d}=0 \end{array}$$

where ${\left\|\cdot \right\|}_{*}$ is the nuclear norm, and $\tau {\left\|\varepsilon \right\|}_{2}$ can be considered the error-penalizing term with penalty parameter τ. The aim of (31) is to recover a low-rank Toeplitz matrix with the values ${\langle r_{Vd}\rangle }_{\mathbb{C}d}$, which is close to ${\tilde{r}}_{\mathbb{C}d}$ and is orthogonal to a subspace constructed using $Q_{CS}$ and $J_{d}$. The main drawback of other coarray interpolation algorithms is that the error bound is set blindly when assigning ${\tilde{r}}_{\mathbb{C}d}$ to ${\langle r_{Vd}\rangle }_{\mathbb{C}d}$; the error may vary a lot according to the environment, and improper setting may deteriorate the matrix completion performance. Our algorithm avoids this problem by estimating the error automatically, and the existence of the error also allows ${\langle r_{Vd}\rangle }_{\mathbb{C}d}$ to satisfy the orthogonal constraint, which is constructed by exploring the additional structure of ${\langle r_{Vd}\rangle }_{\mathbb{C}d}$ and enables our algorithm to obtain a more accurate estimate. The penalty parameter τ can tradeoff the influence of the low-rank term and the error term, which can be set empirically according to the environment: When the signal to noise ratio (SNR) is high and the number of snapshots is large enough, we can set a large τ to penalize the error term; otherwise, a small value is set for τ in pursuit of a solution with a smaller nuclear norm. However, τ is set in a short interval in the simulations as it is not very sensitive to the environment.

Now we turn to the sum coarray model for interpolation, the CCCM of the ULA possesses a Hankel structure according the expression in (7), which means that each ascending skew-diagonal from left to right is constant. The autocorrelation vector on the uniform gird $V_{s}$ is denoted by $r_{Vs}$, which can be considered as the diagonal elements of the Hankel matrix (denoted as $ℋ(r_{Vs})$). The sampling autocorrelation vector on the nonuniform grid ${\mathbb{C}}_{s}$ is obtained by ${\tilde{r}}_{\mathbb{C}s}=J_{s}^{†}\mathrm{vec}({\tilde{R}}_{xx^{*}}^{\alpha }(\tau ))$ according to Equation (13). The sum coarray interpolation problem is similar to the difference coarray interpolation case, and can be formulated as

(32) $$\begin{array}{ll} \min & {\left\|ℋ(r_{Vs})\right\|}_{*}+\tau {\left\|\varepsilon \right\|}_{2}\\ s.t. & {\langle r_{Vs}\rangle }_{\mathbb{C}s}-{\tilde{r}}_{\mathbb{C}s}=\varepsilon \\ & (I-Q_{CCS}Q_{CCS}^{†})J_{s}{\langle r_{Vs}\rangle }_{\mathbb{C}s}=0. \end{array}$$

The minimization functions and the constraints in problems (31) and (32) are convex, so we resort to the cvx toolbox [38] to solve the constrained minimization problems. After $T(r_{Vd})$ or $ℋ(r_{Vs})$ is obtained, the singular value decomposition (SVD) is then implemented to obtain the noise subspace, and subspace-based methods can be used to estimate the DOAs.

Remarks:

1. Even though the recovered matrices $T(r_{Vd})$ and $ℋ(r_{Vs})$ can be considered as the correlation matrices of a ULA with N=(|V|+1)/2 sensors, the DOFs cannot achieve N−1 due to the fact that the filled lags do not provide additional information on the sources. The actual freedom is governed by the nonuniform grid ${\mathbb{C}}_{d(s)}$. For the coprime array illustrated in Figure 1, the numbers of the nonuniform grids for the difference coarray and the sum coarray are $\left|{\mathbb{C}}_{d}\right|=43$ and $\left|{\mathbb{C}}_{s}\right|=35$, respectively. Thus, for a modulated signal that has both the cyclostationarity and the conjugate cyclostationarity property, the difference coarray model-based Toeplitz completion algorithm can resolve more sources than the sum coarray model-based Hankel completion algorithm.
2. The dimensions of the (conjugate) correlation subspaces need to be set carefully. When there is no prior knowledge of the sources, we set the angle interval to [−π/2,π/2], the dimension of the correlation subspace is chosen as $\left|{\mathbb{C}}_{d}\right|$, and $\left|{\mathbb{C}}_{s}\right|$ is set as the dimension of the conjugate correlation subspace. When we have prior knowledge of the sources, we can implement the eigenvalue decomposition (EVD) of the (conjugate) correlation subspace matrices, and the dimensions are set according to the numbers of the resultant positive eigenvalues.
3. The proposed coarray interpolation algorithms are not only suitable for the coprime array, but are also suitable for other partial augmentable arrays as it satisfies the recovery condition of structured matrix completion revealed in [31].
4. The correlation subspace matrix (23) and the conjugate correlation subspace matrix (24) cannot be calculated analytically due to the fact they are unintegrable with respect to $\theta_{i}$. For a symmetric source interval [−β/2, β/2], the following numerical approximations are used instead:
  (33) $$M_{CS}=\sum_{l=-(N-1)\beta /2N}^{(N-1)\beta /2N}\left[\alpha^{*}(l)\alpha^{T}(l)]⊗[\alpha (l)\alpha^{H}(l)\right]/N,$$
  (34) $$M_{CCS}=\sum_{l=-(N-1)\beta /2N}^{(N-1)\beta /2N}\left[\alpha (l)\alpha^{H}(l)]⊗[\alpha (l)\alpha^{H}(l)\right]/N,$$
  where the number of discrete samples is set to N=1001 in the simulation. Even though we use all possible spatial grids to construct the (conjugate) correlation subspaces, this doesn’t affect the fact that our algorithms can resolve gridless sources, which is the advantage over the sparsity-aware algorithms.

The entire process of the proposed algorithms is summarized in Algorithm 1.
**Algorithm 1.** The proposed improved coarray interpolation algorithms with cyclostationary signals.**Input**The received signal vector $\tilde{x}(t)$, cyclic frequency α, sources prior**Output**The optimized low-rank (conjugate) correlation matrix**Step 1**Compute the sampling (conjugate) correlation matrix ${\tilde{R}}_{xx^{\left(*\right)}}^{\alpha }(\tau )$**Step 2**Generate the transform matrix $J_{d(s)}$ and reshape ${\tilde{R}}_{xx^{\left(*\right)}}^{\alpha }(\tau )$ to get ${\tilde{r}}_{\mathbb{C}d(s)}$**Step 3**Construct the (conjugate) correlation subspace $Q_{CS}$ or $Q_{CCS}$**Step 4**Optimize (31) for the difference coarray or (32) for the sum coarray


## 4. Simulation Results

In our simulations, the coprime array was a sparse array with ten sensors located at S={0,3,5,6,9,10,12,15,20,25}, which is illustrated in Figure 1. After the holes are filled, the partial augmentable array can be considered as a ULA with N=26 elements for both the difference coarray model and the sum coarray model. The performance of the proposed coarray interpolation algorithms for the difference coarray model (CI-DC) and the sum coarray model (CI-SC) were compared with several modern algorithms exploiting the coprime array, including the spatial smoothing MUSIC (SS-MUSIC) [22] and the sparsity-aware algorithms for the difference coarray model (SA-DC) and the sum coarray model (SA-SA) [20]. The root mean squared error (RMSE) was used to measure the performance, and is defined as $\mathrm{RMSE}={\left(\sum_{i=1}^{I}\sum_{k=1}^{D}{\left({\hat{\theta }}_{k}-\theta_{k}\right)}^{2}/ID\right)}^{1/2}$, where ${\hat{\theta }}_{k}$ denotes the estimated DOA of the kth source and I=200 is the number of independent repetitions. The sources were binary phase shift keying (BPSK)-modulated including the signals of interest (SOIs) with a 4 Mb/s bit rate and interference with a 3.2 Mb/s bit rate. The proposed algorithms and the sparsity-aware algorithms exploiting cyclic statistics were under the optimal parameters (τ=0.125μs, α=4MHz). Cyclic MUSIC [9] was utilized in our algorithms after the low-rank (conjugate) correlation matrices were obtained.

**Figure 3:**
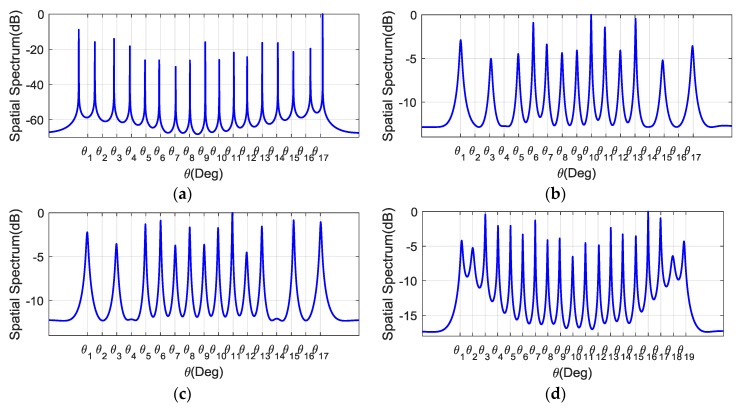
Spatial spectrum of the proposed algorithms and the SS-MUSIC algorithm. Subfigures (**a**–**c**) depict the performance in the first scenario with 13 signals of interest (SOIs) and 4 interference sources; subfigure (**d**) depicts the performance in the second scenario with 19 SOIs. (**a**) SS-MUSIC; (**b**) CI-DC; (**c**) CI-SC; (**d**) CI-DC.

In the first experiment, we tested the interference elimination capacity and the increased DOFs of the proposed algorithms. In the first scenario, D=17 equal-power signals including 13 SOIs and 4 interference sources impinged on the array. All the sources were uniformly distributed between $-60^{\circ }$ and $60^{\circ }$. The interferences arrived from $\left\{\theta_{2},\theta_{4},\theta_{14},\theta_{16}\right\}$. The true DOAs, including the SOIs and the interference sources, are illustrated by the vertical dashed lines in Figure 3a–d. Two thousand snapshots were sampled at the frequency of 32 MHz. The signal-to-noise ratio and the interference-to-noise ratio were 0 dB. The source angle interval was set to [−π/2,π/2]. The eigenvalues of $M_{CS}$ and $M_{CCS}$ are presented in Figure 2. We did not take the sparsity-aware algorithm into account due to its similar performance with our algorithm. Figure 3a–c demonstrate the performance in terms of the spatial spectrum of the SS-MUSIC, CI-DC, and CI-SC algorithms in the first scenario. It should be mentioned that the consecutive range of the difference coarray was from −17 to 17, indicating that the maximal DOF for the SS-MUSIC is 17 under the array configuration of S. It can be seen from Figure 3a that the SS-MUSIC method does not have the signal-selective capacity because it forms peaks at the angles of the interference sources and it has large pointing errors at $\theta_{1}$ and $\theta_{2}$. The proposed CI-DC in Figure 3b and the CI-SC in Figure 3c, as expected, can null out the four DOAs of the interference sources and have correctly determined all the true DOAs of SOIs. In the second scenario, we increased the number of SOIs from D=13 to D=19. The sources were uniformly distributed between $-60^{\circ }$ and $60^{\circ }$. The SNR was 10 dB and 2000 snapshots are sampled. We tested the increased DOF of the proposed CI-DC algorithm shown in Figure 3d. The reason why we choose CI-DC is that the difference-coarray-based algorithm has more nonuniform grids than the sum coarray model; thus, it can resolve more sources than CI-SC. Figure 3d shows that the CI-DC algorithm can correctly estimate all the 19 SOIs and the achieved DOF is beyond the limit of SS-MUSIC, which can only utilize the consecutive part of the difference coarray.

**Figure 4:**
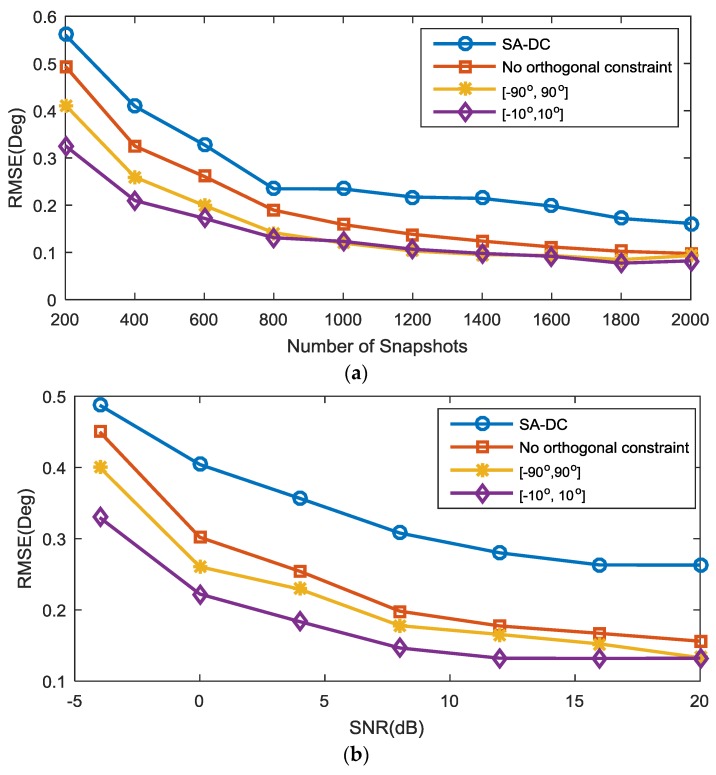
Root mean squared error (RMSE) performance comparison with the difference coarray model. (**a**) RMSE versus the number of snapshots with SNR = 0 dB; (**b**) RMSE versus SNR with 400 available snapshots.

In the second experiment, we compared the RMSE versus the number of snapshots and SNR with the difference coarray model between our CI-DC algorithm (including two different source interval conditions and without the orthogonal constraint condition) and the SA-DC algorithm. Both algorithms utilize the CCM as the second-order statistics and have interference elimination capability. The prespecified grids for SA-DC are from $\left[-90^{\circ },90^{\circ }\right]$ with a sampling interval of $0.1^{\circ }$. The regularization parameter for SA-DC was empirically chosen to be 0.4, and the penalty parameter τ in our algorithm was set to 24 when the number of snapshots is less than 1000 in Figure 4a or the SNR is less than 12 dB in Figure 4b; otherwise, τ was set to 26. Five equal-power BPSK SOIs uniformly distributed between $-9^{\circ }$ and $9^{\circ }$ impinged on the array. In Figure 4a, the SNR is fixed at 0 dB, and the number of snapshots is fixed at 400 in Figure 4b. The other settings were the same as those in the first experiment. As can be seen from Figure 4a,b, even though the SA-DC algorithm can utilize the whole difference coarray information, its performance is inferior to the proposed algorithm due to the basis mismatch caused by the prespecified grids. The proposed CI-DC algorithm with source interval $\left[-10^{\circ },10^{\circ }\right]$ outperforms the condition with source interval $\left[-90^{\circ },90^{\circ }\right]$ and the case without the orthogonal constraint, especially when the SNR is low and the snapshots are limited; this indicates that a smaller source interval helps to improve the performance remarkably. When the number of snapshots is larger than 1000 in Figure 4a and the SNR is larger than 16 dB in Figure 4b, the RMSE of the proposed algorithm with different constraint conditions is similar.

**Figure 5:**
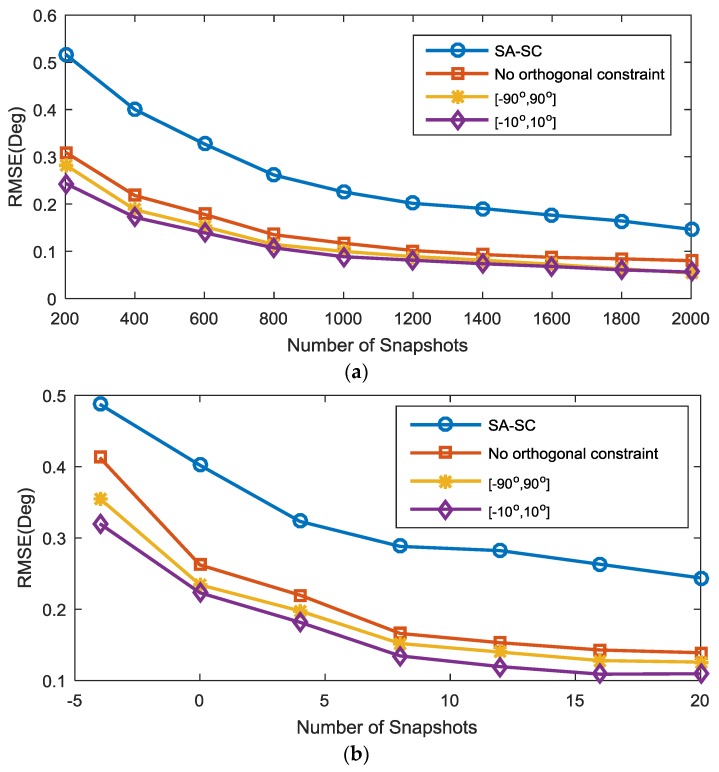
RMSE performance comparison with the sum coarray model. (**a**) RMSE versus the number of snapshots with SNR = 0 dB; (**b**) RMSE versus SNR with 400 available snapshots.

In the third experiment, we compared the RMSE versus the number of snapshots and the SNR with the sum coarray model between our algorithm with three different constraint conditions and the SA-SC algorithm. The sources and the parameters in both algorithms were the same as those in the second experiment. It can be seen from Figure 5 that the CI-SC with a small source interval performs the best, which indicates that the orthogonal constraint incorporated with a smaller source interval helps to regulate the estimate toward a more accurate solution. This observation coincides with the result in the second experiment.

In the last experiment, we intended to resolve more sources than a single coarray interpolation scheme by utilizing both the obtained $T(r_{Vd})$ and $ℋ(r_{Vs})$. We used the extended cyclic MUSIC method [10] and simply constructed the extended cyclic correlation matrix as follows:

(35) $$R_{CE}=\left[\begin{array}{cc} T(r_{Vd}) & ℋ(r_{Vs})\\ {ℋ}^{*}(r_{Vs}) & T^{*}(r_{Vd}) \end{array}\right].$$

**Figure 6:**
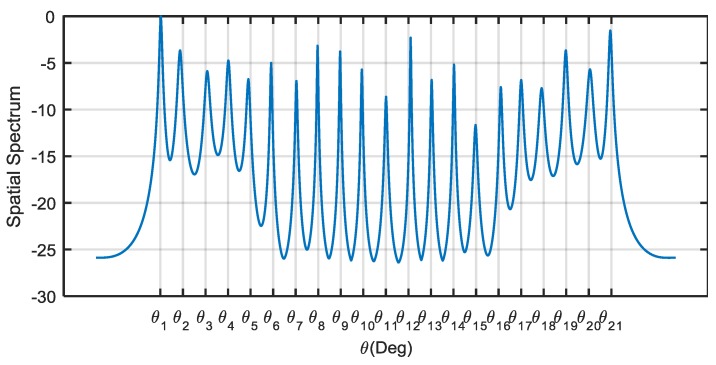
The spatial spectrum of the extended cyclic MUSIC method with 21 sources.

Then, the SVD of $R_{CE}$ was implemented to obtain the noise subspace $U_{n}$, and the spatial spectrum of the extended cyclic MUSIC method was given by

(36) $$P(\theta )=\frac{1}{\alpha^{H}(\theta )U_{n1}U_{n1}^{H}\alpha^{H}(\theta )-\left\|\alpha^{T}(\theta )U_{n2}U_{n1}^{H}\alpha^{H}(\theta )\right\|}$$

where $U_{n}=[U_{n1};U_{n2}]$ is the noise subspace, and $U_{n1}$ and $U_{n2}$ are two submatrices of the same dimension. The spectrum searching scheme was utilized to find all the peaks. Twenty-one equal-power BPSK SOIs uniformly distributed between $-70^{\circ }$ and $70^{\circ }$ impinged on the same coprime array as specified in Figure 1. The SNR was set to 20 dB and the number of snapshots was 2000. The other parameters were set to be the same as those in the first experiment. After the CI-DC and CI-SC algorithms were completed, the SVD of $R_{CE}$ was implemented to obtain the noise subspace and the spatial spectrum was calculated based on (36). The spatial spectrum of the extended cyclic MUSIC method is illustrated in Figure 6. It can be seen that all the 21 SOIs were correctly determined using the extended cyclic MUSIC method; this result validates the hypothesis that the extended cyclic MUSIC method, utilizing the solutions from the structured matrix completion, can achieve more DOFs than the single coarray interpolation scheme. However, rather than simply constructing the extended cyclic matrix with the respective solutions from Toeplitz completion and Hankel completion, there is potential to resolve more sources by directly solving the extended cyclic correlation matrix completion problem with structure as in (35).

## 5. Conclusions

In this paper, improved coarray interpolation algorithms were proposed for cyclostationary signals. We integrate the cyclostationarity with the spatial domain and performed DOA estimation under the circumstance that the sources are more numerous than the sensors. By exploring the structure of the CCM and the CCCM in a coprime array, we built up the difference coarray and sum coarray models and formulated the coarray interpolation as a Toeplitz completion problem and a Hankel completion problem. In order to further improve the performance of the coarray interpolation, we defined the spatial spectrum sampling operators and the derivative (conjugate) correlation subspaces, then constructed orthogonal constraints for the autocorrelation vectors in the structured matrix completion problems. Prior knowledge of the source intervals was also incorporated into the problem, which helped to improve the performance significantly. Numerical results validated the effectiveness of the proposed algorithms and demonstrated their superiority in terms of interference elimination capacity, increased DOFs, and estimation accuracy.

## Appendix A

In this appendix, we prove that for a ULA with P physical sensors, the P-points averaged inner product of the two steering vectors $\alpha^{H}(\theta_{i})\alpha (\theta_{k})/P$ ($\theta_{i},\theta_{k}\in [-\pi /2,\pi /2]$) can be approximated by a Kronecker delta function when P is large enough. The steering vector of a ULA with P physical sensors can be expressed as

(A1) $$\alpha (\theta )={\left[1,\;e^{j\pi \sin\theta },\ldots e^{j\pi (P-1)\sin\theta }\right]}^{T}.$$

Then the P-points averaged inner product of the two steering vectors is given by

(A2) $$f(\theta_{i},\theta_{k})=\frac{1}{P}\alpha^{H}(\theta_{i})\alpha (\theta_{k})=\frac{1}{P}\sum_{n=0}^{P-1}e^{jn\pi (\sin\theta_{k}-\sin\theta_{i})}.$$

Let $x=\frac{P}{2}(\sin\theta_{k}-\sin\theta_{i})$, then (A2) can be rewritten as $f(x)=\frac{1}{P}\sum_{n=0}^{P-1}e^{j(2\pi /P)nx}$, and f(x) can be seen as a time-domain signal corresponding to a P-points discrete rectangular function in the frequency domain. Thus, we can obtain

(A3) $$f(x)=\frac{1}{P}\frac{\sin(\pi x)}{\sin(\pi x/P)}e^{j(\frac{P-1}{P})\pi x}.$$

When P is large enough, f(x) will be approximated as

(A4) $$f(x)\approx \delta_{i,k}=\left\{ \begin{array}{ll} 1, & i=k\\ 0, & i\neq k \end{array}\right..$$

Consequently, we can draw the conclusion that $f(\theta ,\theta_{0})$ will approximate a Kronecker delta function when P is large enough. However, for a nonuniform linear array, we can still approximate $f(\theta_{i},\theta_{k})$ with a Kronecker delta function due to the fact that $f(\theta_{i},\theta_{k})$ with $\theta_{i}=\theta_{k}$ is much larger than the values when $\theta_{i}\neq \theta_{k}$.
